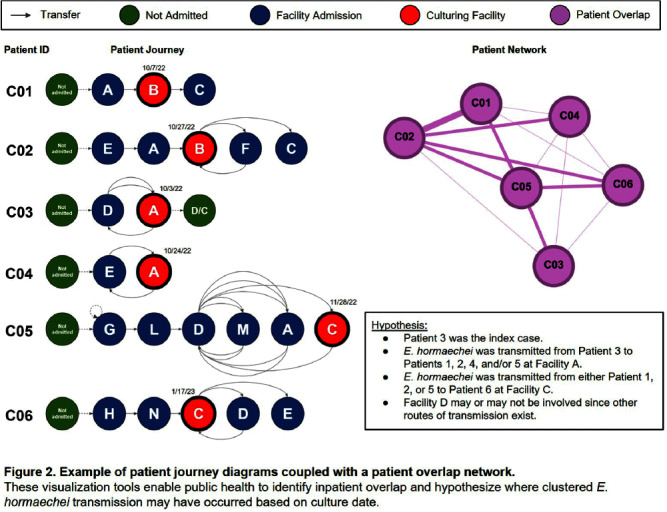# Public Health Applications of Patient Transfer Networks—Colorado, 2022–2023

**DOI:** 10.1017/ash.2024.118

**Published:** 2024-09-16

**Authors:** Kristen Marshall, Karlie Hoetzer, Jennifer Driscoll, Braden Bardach, Janell Nichols, Samuel Baird, Vishnu Panicker, Devon Williford, Christopher Czaja

**Affiliations:** CDC; Colorado Department of Public Health and Environment; Ascension All Saints Hospital; Colorado Department of Public Health

## Abstract

**Background:** During 2021–2023, an increase in Klebsiella pneumoniae carbapenemase producing Enterobacterales species (KPC-CRE) cases occurred among patients admitted to several overlapping healthcare facilities, prompting an investigation by the Colorado Department of Public Health and Environment (CDPHE). We applied social network analysis (SNA) to identify KPC-CRE networks and other multidrug-resistant organism (MDRO) transmission, created a tool for public health prevention planning and for facilities to examine their own patient transfer connectivity, and explored additional public health and emergency preparedness applications. **Methods:** A statewide patient transfer network was created using 2021–2022 Medicare beneficiary data. Sub-networks were isolated from the larger network to examine a cluster of facilities involved in a KPC-CRE outbreak, defined as ≥2 KPC-CRE cases related by whole genome sequencing (WGS). WGS was conducted at the CDPHE State Lab. Highly connected facilities were determined by patient transfers between at least two KPC-CRE testing facilities. Individual patient journeys were constructed using admissions and culture date. SNA was conducted in RStudio; visualizations, network metric calculations, and clustering analysis were conducted using Gephi and ArcGIS software. **Results:** SNA yielded 4,864 direct patient transfers between 326 healthcare facilities (220 skilled nursing facilities, 50 acute care hospitals, 32 critical access hospitals, six long term acute care hospitals, and 18 facilities not previously classified; Figure 1). WGS identified five separate KPC-CRE outbreaks among 14 patients during February 2022–January 2023; 14 patient specimens were collected at four testing facilities. We identified five highly connected facilities in addition to the four testing facilities. Patient journeys allowed us to identify possible locations of KPC-CRE transmission in four of the five outbreaks (Figure 2). CDPHE provided guidance to all involved facilities on admission screening, routine point prevalence surveys, and interfacility communication as part of an MDRO prevention plan. CDPHE then developed the transfer network into an interactive ArcGIS dashboard enabling facilities to examine their own patient transfer patterns. **Conclusions:** SNA enabled CDPHE to identify at-risk facilities for KPC-CRE transmission and create an interactive tool for facility and public health use. Future applications of patient transfer networks can include geographical grouping of facilities based on transfers to zone healthcare coalitions and conduct preparedness activities, and creating medical operations preparedness plans for emergencies or disasters.

**Figure:**
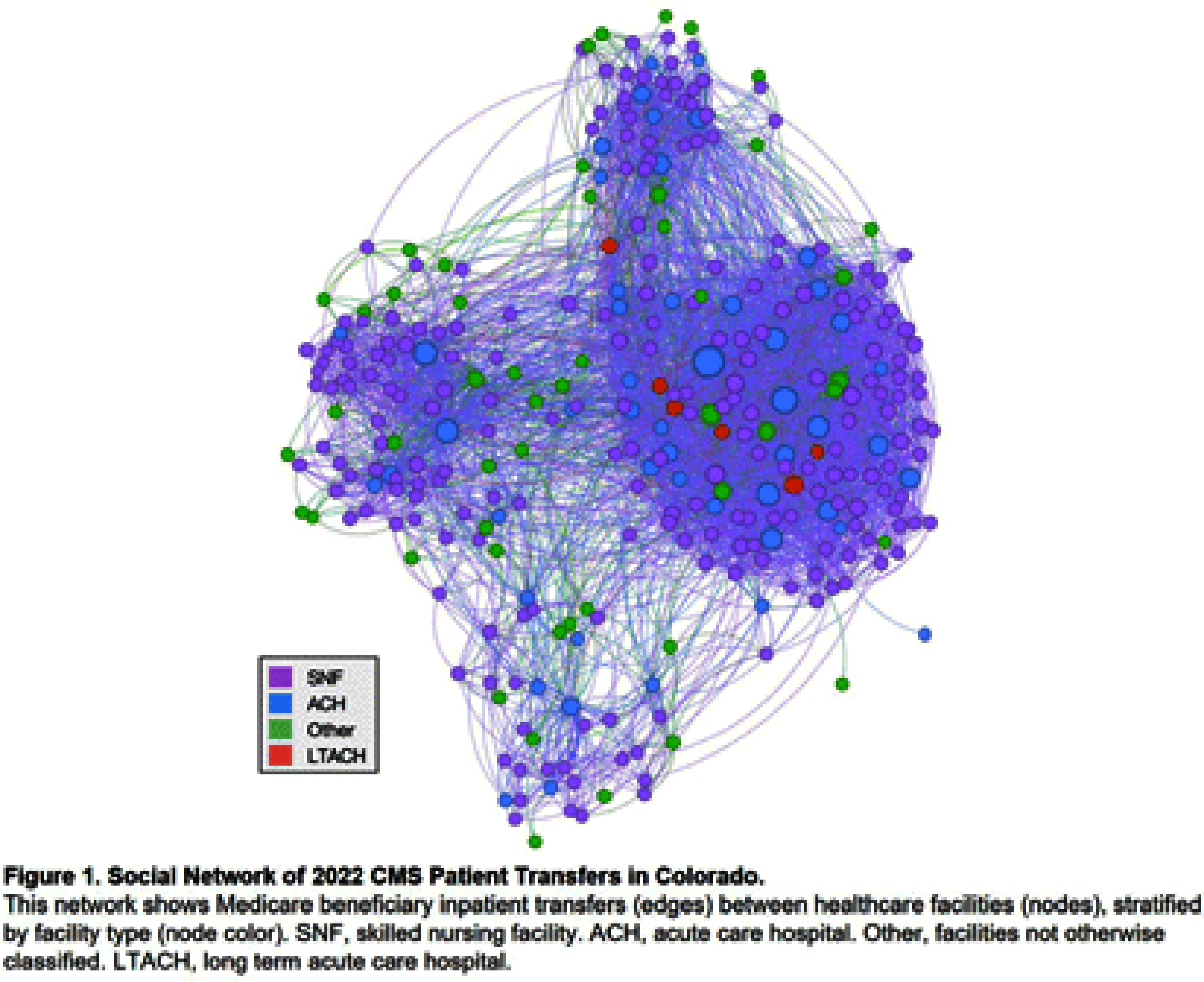

**Figure:**